# A phase I trial of the selective oral cyclin-dependent kinase inhibitor seliciclib (CYC202; *R*-Roscovitine), administered twice daily for 7 days every 21 days

**DOI:** 10.1038/sj.bjc.6603509

**Published:** 2006-12-19

**Authors:** C Benson, J White, J De Bono, A O'Donnell, F Raynaud, C Cruickshank, H McGrath, M Walton, P Workman, S Kaye, J Cassidy, A Gianella-Borradori, I Judson, C Twelves

**Affiliations:** 1 Cancer Research UK Centre for Cancer Therapeutics, The Institute of Cancer Research, Sutton, Surrey, UK; 2 Section of Medicine, The Institute of Cancer Research and Royal Marsden Hospital, Sutton, Surrey, UK; 3 Department of Medical Oncology and Beatson Oncology Centre, University of Glasgow, Glasgow, UK; 4 Cancer Research UK Drug Development Office, London, UK; 5 Cyclacel Ltd, Dundee, UK; 6 Beatson Oncology Centre, Glasgow, UK

**Keywords:** cyclin-dependent kinase inhibitor, phase I clinical trial

## Abstract

Seliciclib (CYC202; *R*-roscovitine) is the first selective, orally bioavailable inhibitor of cyclin-dependent kinases 1, 2, 7 and 9 to enter clinical trial. Preclinical studies showed antitumour activity in a broad range of human tumour xenografts. A phase I trial was performed with a 7-day b.i.d. p.o. schedule. Twenty-one patients (median age 62 years, range: 39–73 years) were treated with doses of 100, 200 and 800 b.i.d. Dose-limiting toxicities were seen at 800 mg b.i.d.; grade 3 fatigue, grade 3 skin rash, grade 3 hyponatraemia and grade 4 hypokalaemia. Other toxicities included reversible raised creatinine (grade 2), reversible grade 3 abnormal liver function and grade 2 emesis. An 800 mg portion was investigated further in 12 patients, three of whom had MAG3 renograms. One patient with a rapid increase in creatinine on day 3 had a reversible fall in renal perfusion, with full recovery by day 14, and no changes suggestive of renal tubular damage. Further dose escalation was precluded by hypokalaemia. Seliciclib reached peak plasma concentrations between 1 and 4 h and elimination half-life was 2–5 h. Inhibition of retinoblastoma protein phosphorylation was not demonstrated in peripheral blood mononuclear cells. No objective tumour responses were noted, but disease stabilisation was recorded in eight patients; this lasted for a total of six courses (18 weeks) in a patient with ovarian cancer.

The cyclin-dependent kinases (Cdks) have been reported to play critically important roles in several key cellular processes that are frequently dysregulated in cancer, including cell cycling, transcription and apoptosis (Malumbres and Barbacid, 2001; Benson *et al*, 2005). The design of small molecule inhibitors of Cdks has, therefore, been actively pursued, and a considerable number of small molecule inhibitors of these serine–threonine kinases have been identified. The first compounds in this class to be evaluated in clinical trials were flavopiridol and the 7-hydroxy derivative of staurosporine, UCN-01 (Senderowicz *et al*, 1998; Sausville *et al*, 2001). These first-generation Cdk inhibitors lacked specificity, being broad-spectrum inhibitors that inhibited not only the Cdks but also many other kinases. Second-generation inhibitors were designed to be more selective, with many of these compounds specifically developed to target selected Cdks (Meijer and Raymond, 2003). Seliciclib (CYC202; *R*-roscovitine) is a 2,6,9-tri-substituted purine analogue of olomoucine derived from studies evaluating the structure–activity relationships within this compound class (Meijer *et al*, 1997; Gray *et al*, 1998; McClue *et al*, 2002; Whittaker *et al*, 2004). The co-crystal structure of seliciclib and Cdk2 has been described, with the drug occupying the ATP-binding pocket of the kinase (De Azevedo *et al*, 1997).

Seliciclib is a highly selective, orally bioavailable, small molecule inhibitor of several Cdks, competing at their ATP-binding sites (Meijer *et al*, 1997). It is a potent inhibitor of the human Cdk2/cyclin E, Cdk1/cyclin B, Cdk7/cyclin H and Cdk9/cyclin T1 with IC_50_s of 0.1, 2.7, 0.5 and 0.8 *μ*M, respectively (McClue *et al*, 2002). It is, however, only a weak inhibitor of Cdk4, Cdk6 and Cdk8. In a 148 kinase screen, seliciclib was highly Cdk specific, with few other enzymes being inhibited only at micromolar concentrations (Bach *et al*, 2005). The latter includes CaM kinase 2, CK1*α*, CK1*δ*, DYRK1A, EPHB2, ERK1, ERK2, FAK and IRAK4. The other kinases tested were insensitive to seliciclib. This screen was not, however, exhaustive, testing only approximately 30% of known kinases. Affinity purification of proteins bound to *R*-roscovitine-agarose beads also identified pyridoxal kinase (PDXK) as a target of seliciclib, binding not at the ATP-binding site but at the pyridoxal site (Tang *et al*, 2005). Pyridoxal kinase (PDXK) catalyses the phosphorylation of pyridoxine, pyridoxal and pyridoxamine to, respectively pyridoxine-5′-phosphate, pyridoxal-5′-phosphate and pyridoxamine-5′-phosphate respectively.

Seliciclib has antitumour activity against a broad range of cancer cell lines (McClue *et al*, 2002; Whittaker *et al*, 2004; Raynaud *et al*, 2005). Molecular pharmacology studies in human colorectal cancer cell lines have revealed that seliciclib decreases retinoblastoma protein phosphorylation, initially at the Cdk2 phosphorylation site and then at multiple sites (Whittaker *et al*, 2004). Seliciclib also causes increases in Erk-1 and Erk-2 phosphorylation, although this may have little impact on cell cycling (Whittaker *et al*, 2004). More importantly, through its inhibitory effects on Cdk7 and Cdk9, seliciclib inhibits RNA polymerase II phosphorylation and total RNA polymerase II protein activity (Whittaker *et al*, 2004; Raynaud *et al*, 2005). This results in the decreased expression of several cyclins, including cyclins D1, A and B1, which may explain the observed global effects on retinoblastoma (RB) phosphorylation and the overall cell cycle effects of seliciclib in these models. These include a decreased proportion of cells in G1, decreased DNA synthesis in the S-phase and a moderate increase in cells in G_2_/M (Whittaker *et al*, 2004; Raynaud *et al*, 2005).

Other studies report that this agent also blocks the degradation of p53 through the inhibition of MDM2 expression (Lu *et al*, 2001). Studies in the Lovo colorectal carcinoma cell line indicate that the major effect on these cells is the induction of cell death from all compartments of the cell cycle (Schutte *et al*, 1997; McClue *et al*, 2002). The antitumour efficacy of seliciclib has been demonstrated in human tumour xenografts in nude mice, when administered at 500 mg kg^−1^ twice a day or 200 mg kg^−1^ thrice a day (McClue *et al*, 2002; Raynaud *et al*, 2005). Pharmacokinetic–pharmacodynamic relationships were established in the HCT116 human colon cancer xenograft model and showed reduced phosphorylation of RB and decreased expression of cyclin D1 in tumour tissue (Raynaud *et al*, 2005). Seliciclib was most active in inhibiting the proliferation of colon, non-small-cell lung, breast and prostate human cancer xenografts. It also induces caspase-dependent apoptosis in primary B-cell chronic lymphatic leukaemia cells *ex vivo*, downregulating the antiapoptotic proteins Mcl-1 and XIAP, and increasing Bak expression and Bax cleavage (Alvi *et al*, 2005). In addition, seliciclib demonstrates antitumour activity in multiple myeloma cultures and cell lines, inducing apoptosis associated with downregulation of Mcl-1 and interleukin (IL)-6 transcription and protein expression (MacCallum *et al*, 2005; Raje *et al*, 2005).

Seliciclib has a satisfactory preclinical pharmacokinetic (PK) profile and is extensively cleared into inactive metabolites, with the carboxylate being the main urinary metabolite after administration to mice and following incubation with hepatic microsomes (Nutley *et al*, 2005). Seliciclib demonstrated a higher area-under-the-curve (AUC), longer elimination half-life and better intratumoral drug delivery, when compared with the similar 2,6,9-tri-substituted purine Cdk inhibitors olomoucine and bohemine (Raynaud *et al*, 2004). Seliciclib is highly bound to plasma proteins (90%), primarily albumin (Vita *et al*, 2005). To characterise further the PK of this agent, a first in-human, single oral dose, volunteer study was undertaken in 12 healthy subjects, administering between 50 and 800 mg of seliciclib (De la Motte and Gianella-Borradori, 2004). This showed that the parent compound is satisfactorily absorbed, undergoes first-pass metabolism, exhibits high protein binding and is rapidly and extensively distributed into tissues. The carboxylate was confirmed as the main metabolite with low protein binding, limited tissue distribution and renal clearance.

In view of these promising data, seliciclib has been studied in early clinical trials in patients with advanced cancer, utilising several schedules to further evaluate its safety, tolerability and PK properties. The principal objectives of this phase I trial of seliciclib administered orally twice a day for 7 days every 21 days were (1) to determine the toxicity profile of seliciclib (2) to identify the dose limiting toxicities of seliciclib, (3) to establish the recommended dose of seliciclib on this schedule, (4) to characterise the PK behaviour of seliciclib and (5) to document possible anticancer activity in patients with advanced solid malignant diseases.

## PATIENTS AND METHODS

### Patient selection

Patients with a histologically confirmed diagnosis of a malignant solid tumour, refractory to conventional treatment were eligible for treatment, provided they met the following criteria: age ⩾18 years; World Health Organization (WHO) performance status⩽2; estimated life expectancy ⩾3 months; no previous anticancer therapy within 4 weeks; no significant gastrointestinal disease influencing drug absorption; adequate hematopoietic, hepatic and renal functions (Hb⩾9 g dl^−1^, WBC⩾3.5 × 10^9^ l^−1^, neutrophils>1.5 × 10^9^ l^−1^, platelets⩾100 × 10^9^ l^−1^, bilirubi⩽1.25 × upper limit of normal and serum aspartate aminotransferase, alanine aminotransferase, and alkaline phosphatase<2.5 the upper limit of normal, plasma creatinine ⩽130 *μ*mo l^−1^ or calculated creatinine clearance according to the Cockroft formula ⩾60 ml min^−1^). The study was managed and monitored by the Cancer Research UK Drug Development Office and conducted according to Good Clinical Practice (GCP) at the Royal Marsden Hospital (Sutton) and the Beatson Oncology Centre (Glasgow). The protocol was reviewed by the Cancer Research UK Central Institutional Review Board (CIRB) protocol review committee and the clinical research ethics committees at the above centres. All patients gave written informed consent before entry to the study.

### Treatment and dose escalation

Seliciclib was supplied by Cyclacel Ltd (Dundee, UK) as 50 and 200 mg capsules, stored at room temperature. Capsules were taken twice daily with the patient fasting 2 h before and after drug dosing. Patients were asked to record daily the number of capsules taken, the time of intake and also when they had last eaten. Compliance was assessed by counting the number of capsules returned by the patient at the end of each treatment cycle. Patients were hospitalised for the first cycle of treatment to facilitate toxicity monitoring and PK sampling. Thereafter, they were treated on an outpatient basis.

The starting dose of seliciclib was selected on the basis of animal toxicology and PK experiments and a single dose study in patients over the dose range 50–200 mg. A dose of 400 mg (250 mg m^−2^) was predicted to achieve meaningful plasma concentrations (>10 *μ*M) based on preclinical PK data, and a single-dose human study demonstrated that the elimination half-life was suitable for twice daily administration, although the interindividual variability in clearance was high (De la Motte and Gianella-Borradori, 2004). It was therefore decided to start at 100 mg P.O. twice daily (b.i.d.) to be administered for 7 days every 3 weeks. At least three patients were treated at each dose level. Planned dose increments were at least 100% until significant drug-related toxicity was observed (grade 1 non-haematological or grade 2 neutropenia or thrombocytopenia), and 40% thereafter.

Dose-limiting toxicity (DLT) was defined as drug-related toxicity in the first cycle, including grade 4 neutropenia for 7 days or more, neutropenic fever, platelet count <25 × 10^9^ l^−1^ and/or non-haematological toxicity⩾grade 3. This definition excluded nausea and vomiting subsequently responding to antiemetic therapy and reversible grade 3 increases in liver transaminases. The maximum tolerated dose (MTD) was defined as a dose below which DLT occurred in more than 30% of the patients. Toxicities were evaluated according to the National Cancer Institute Common Toxicity Criteria, version 2.0.

### Treatment assessment

Before commencing treatment, a complete medical history was taken and physical examination was performed. The following pretreatment baseline examinations were performed within 4 weeks of starting treatment: full blood count, including white blood cell differential; reticulocyte count, haptoglobin, prothrombin time and Coombs test; serum biochemistry, including sodium, potassium, urea, creatinine, calcium, phosphorus, creatine kinase and isoenzymes, total protein, albumin, bilirubin (direct and total), alkaline phosphatase (ALP), alanine aminotransferase (ALT), aspartate aminotransferase (AST), *γ*-glutamyl transferase (*γ*GT), lactate dehydrogenase and uric acid; urinalysis; pregnancy test, electrocardiogram and a chest X-ray. Weekly evaluations included history, physical examination, toxicity assessment according to the CTC criteria version 2.0 and full blood count and serum chemistries. Radiographic assessment of tumour was performed within 3 weeks before the patient starting treatment and after every two cycles, according to standard RECIST methodology. Patients were taken off protocol upon disease progression, unacceptable toxicity, investigator discretion, serious violation of protocol or at their own request, and were followed for 28 days after the last administration of the study drug.

### Sample collection and pharmacokinetic analysis

For PK analysis, blood samples (approximately 8 ml each) were collected in tubes with lithium heparin anticoagulant at the following time points: pre-dose and 0.5, 1, 1.5, 2, 4, 8, 12 (pre-second dose), 13, 14 and 24 h (pre-dose if day 2) after dosing on both days 1 and 7 of the first cycle. The samples were then centrifuged at 3000 r.p.m at 4°C for 10 min, the plasma transferred to 2 ml cryovials and frozen at −70°C until analysis. Urine was collected post-dosing for 24 h on the first day of seliciclib administration, the total urine volume was recorded and an aliquot stored at −20°C for analysis. Concentrations of seliciclib and its principal metabolites in plasma and urine were determined according to a validated liquid chromatography–mass spectrometry detection method at Quintiles, UK and ACC, Germany.

Pharmacokinetic (PK) analysis was performed using the trapezoidal method and log-linear regression. The following parameters were determined either by calculation or observation: the observed maximal concentration (*C*_max_), the time of observed maximal concentration (*T*_max_), the area under the concentration–time curve calculated by the trapezoidal rule (time 0 to last sample with a quantifiable concentration (AUC _(0−tz)_) and extrapolated to infinity (AUC_(0−∞)_), the terminal rate constant, by log-linear regression (*λ*_z_) and the terminal half-life from *λ*_z._ The value of AUC_(0−∞)_ was considered unreliable if the terminal area beyond the last quantified sample was greater than 20% of the total AUC_(0−∞)._ Pharmacokinetic (PK) data analysis was carried out using a noncompartmental analysis model with the aid of Kinetica (Innaphase Limited, Amersham, UK).

### Sample collection and pharmacodynamic studies

Preclinical studies indicated that inhibition of RB phosphorylation in tumour could be detected at doses associated with inhibition of tumour growth. It was not clear whether surrogate tissues would provide a similar pharmacodynamic (PD) signal. Samples for PD analysis were taken predose and on days 3, 5, 7 and 14 during the first cycle of treatment with seliciclib. Whole blood was collected from patients at selected time points into lithium–heparin Vacutainer^®^ tubes (Vacutainer, BD, Franklin Lakes, NJ, USA). Each tube was inverted several times to ensure thorough mixing and then mixed with sterile 0.9% saline at a ratio of 1:1. This was then layered over ficol (Lymphoprep^®^ Nycomed, Oslo, Norway) and centrifuged at 1200 r.p.m. (400 **g** equivalent) at 18°C for 30 min. The resulting mononuclear cell layer was then removed and resuspended in 0.9% NaCl. Cell pellets were obtained by centrifugation (600 g) and flash frozen in liquid nitrogen. Mononuclear cells were then stored at −70°C in labelled cryovials until further analysis.

Pharmacodynamic analysis was based on molecular markers developed in preclinical models, including the HCT116 human colon tumour xenograft, namely inhibition of RB and RNA polymerase II phosphorylation and downregulation of cyclin D1 (Whittaker *et al*, 2004; Raynaud *et al*, 2005). Cell pellets were treated with lysis buffer (50 mM Tris–HCl, 150 mM NaCl, pH 7.5, 1% NP40, 2 mM PMSF, 10 *μ*g ml^−1^ aprotinin, 10 *μ*g ml^−1^ leupeptin, 1 mM activated sodium orthovanadate, 1 mM sodium fluoride and 10 mM *β*-glycerophosphate) for 30 min on ice. Samples (20–100 *μ*g of protein) were heat-denatured in Laemmli sample buffer (10% glycerol, 5% *β*-mercaptoethanol, 2% sodium dodecyl sulphate (SDS), 62.5 mM Tris (pH 6.8), 0.05% bromphenol blue, final concentrations) and separated by SDS—polyacrylamide gel electrophoresis (PAGE) using pre-cast 10 or 15-well 20 or 10% Tris–glycine polyacrylamide gels (Invitrogen, Netherlands). All constituents of lysis and loading buffers were obtained from Sigma (Poole, UK) The membrane was then blocked with 0.5% casein blocking buffer (0.5% casein hammerstein, 154 mM NaCl, 10 mM Tris base, pH 7.6) incubated overnight with primary antibody (phospho-RB Ser-780: Cell Signalling Technology, MA, USA); total RB protein, sc-50, (Santa Cruz Biotechnology, CA, USA); cyclin A, Ab 6 (Neomarkers, Fremont, CA, USA); Phospho ser5 pol II, R2033-20, US Biological (Swampscott, MA, USA); Phospho ser2 pol II, H5 MMS-129R (Covance, Berkeley, CA, USA); total pol II, Ab5408 (Abcam, Cambridge, UK); and GAPDH (Chemicon, CA, USA).

Visualisation of the bound primary antibody was performed by probing with horseradish peroxide (HRP)-conjugated secondary antibody (anti-rabbit (Bio-Rad, CA, USA); anti-mouse (Amersham Biosciences, Bucks, UK)). The membrane was immersed in ECL reagent (Pierce Biotechnology, Rockford, IL, USA) for 1 min and then exposed to photographic film (Hyperfilm, Amersham Biosciences, Bucks, UK) and processed.

## RESULTS

### General

Between August 2001 and September 2003, 22 patients with metastatic disease were enrolled from the two centres and their demographic characteristics are listed in Table 1. All 22 patients were eligible, but one was withdrawn before receiving treatment owing to rapid disease and symptom progression. Patients had received a median number of two prior chemotherapy regimens. A total number of 42 fully assessable courses were administered. The median number of courses per patient was 2; range 1–6. The following dose levels were studied: 100 mg b.i.d, 200 mg b.i.d and 800 mg b.i.d.

No significant toxicity was seen at the 100 mg dose level at which three patients were treated. At the 200 mg dose level, one patient with advanced colorectal cancer experienced an increase in plasma and liver enzymes, consistent with cholestasis, which did not revert to baseline during the period of the study. The patient's liver biochemistry at baseline was within normal limits but, between days 21 and 28 grade 3 elevations of bilirubin, *γ*GT, ALP, AST and ALT were noted. Subsequent ultrasound investigation revealed new liver metastases, but no features of biliary obstruction. The changes in liver biochemistry persisted and did not revert to baseline upon cessation of treatment, suggesting this may have been tumour-related. The patient was consequently withdrawn from the trial. Nonetheless, this was classified as a DLT, as it was not transient. Three additional patients were recruited at this dose level and no further DLT was reported. At this point, review of the PK data obtained at the 100 and 200 mg dose levels indicated that these doses resulted in very low drug exposure. In addition, 400 mg twice daily for 5 days every 3 weeks appeared to be well tolerated in another seliciclib Phase I trial ongoing at the time. A protocol amendment was made to dose escalate from 200 to 800 mg. At the 800 mg dose level, reversible grade 1 and 2 increases in plasma creatinine, reversible grade 3 and 4 hypokalaemia, reversible grade 3 hyponatraemia and a grade 3 skin rash were observed, so the cohort was expanded to six patients. No further DLTs were experienced in the additional group of three patients. A further protocol amendment allowed a cohort expansion of an additional group of six patients (12 patients in total) to facilitate closer investigation of the putative renal/biochemical toxicities of seliciclib. Four of the final six patients treated at 800 mg experienced seliciclib-related DLTs. In three of these patients, this involved reversible elevations of *γ*GT, and in one patient, hyperglycaemia and glycosuria.

### Toxicity profile

Overall haematological toxicity was mild and did not result in DLT. Nausea was the most commonly reported adverse event and increased in frequency with increasing dose. At the 800 mg dose level, 11 of the 12 patients experienced nausea, although this was not dose-limiting. This symptom was satisfactorily managed with routine antiemetics, including 5-HT_3_ antagonists. Other commonly reported drug-related adverse events included lethargy, fatigue and anorexia. Hypokalaemia, rash and fatigue were the principal DLTs, and were mainly observed at the 800 mg b.i.d. dose level. Further details of these adverse events are summarised in Table 2.

### Electrolyte disturbance and renal impairment

Hypokalaemia considered related to seliciclib was noted in patients receiving 800 mg b.i.d. At this dose, hypokalaemia was observed in five of 12 patients (<3.0 mmol l^−1^), which was grade 1 in two patients, grade 3 in two patients and grade 4 in one patient. This was not deemed to be a result of nausea and vomiting or concomitant diuretic or nephrotoxic therapy. These biochemical changes were first observed on day 7 of seliciclib dosing, as routine electrolyte testing was initially performed only on days 1 and 7 during the treatment period. Additional blood testing revealed that these changes occurred as early as day 3 of treatment. Hypokalaemia was easily reversible with potassium supplementation and was not associated with any electrocardiographic changes. Clinically significant elevations in urea and creatinine were also observed and were correctable with increased oral or intravenous fluids. These biochemical changes appeared to occur separately from the aforementioned hypokalaemia.

This toxicity can be characterised further by describing a typical patient history. In one patient treated at 800 mg b.i.d., serum potassium fell to 2.2 mmol l^−1^ on day 7 of course 1, with a creatinine level just above normal and no other identifiable cause of hypokalaemia. This electrolyte disturbance required hospitalisation, for administration of intravenous and oral potassium supplementation. This patient also developed hypokalaemia after receiving course 2, with a nadir of 2.5 mmol l^−1^, and on this occasion associated with hyponatraemia (127 mmol l^−1^) and hypotension (82/52 mmHg) with a creatinine that was just above the normal range (130 *μ*mol l^−1^; normal range <110 *μ*mol l^−1^). These were, however, possibly associated with concurrent poor oral fluid intake.

At this point, the 800 mg cohort was expanded further to investigate the possible renal effects of seliciclib. More restrictive eligibility criteria were introduced, including a glomerular filtration rate above 60 ml min^−1^ (estimated by ^51^Cr-EDTA) clearance or 24-h urine collection for creatinine clearance) and serum potassium within normal limits. In addition, more intensive blood chemistry testing was performed on days 1, 3, 5 and 7. Furthermore, 24-h urine collections were obtained on days 1 and 7 and analysed for creatinine clearance, urinary electrolytes and urine microscopy. No overall changes were seen in urinalysis, but in one patient with hypokalaemia in this cohort, potassium excretion in the urine was increased on day 7 compared with day 1, suggestive of renal potassium loss.

**Figure 1:**
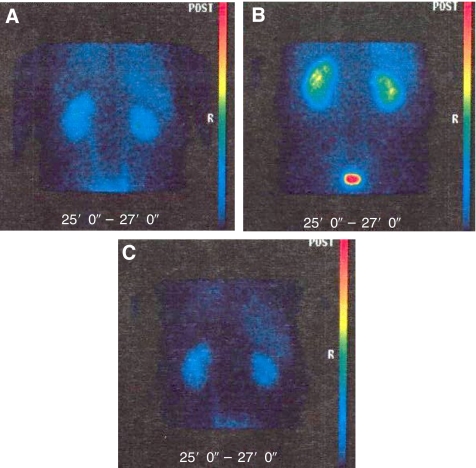
Functional renal perfusion imaging by MAG3 nuclear scanning, performed pretreatment (**A**) and post-treatment on days 3 (**B**) and 14 (**C**), in a patient who experienced a reversible decline in renal function when treated with seliciclib at 1600 mg day^−1^. Baseline pre-treatment renogram (**A**) shows slight asymmetry in renal function with the right kidney (dark green) slightly poorer than the left (light green). Day 3 renogram (**B**) shows changes consistent with bilateral parenchymal retention and delayed intrarenal transit bilaterally, which then almost completely recovers by day 14 (**C**).

In an attempt to understand further the pathogenesis of these biochemical toxicities, three patients were studied more intensively. Urinary retinol-binding protein (RBP), a marker of renal tubular damage was measured in conjunction with functional renal imaging (MAG3 renograms) to estimate renal blood flow pretreatment on days 3 and 14. One patient experienced a rapid, but reversible, increase in creatinine associated with a reduction in renal perfusion bilaterally, determined by MAG3 perfusion indices. This was associated with bilateral increases in renal parenchymal retention on day 3 on MAG3 scans, which reverted to normal by day 14 (see Figure 1). By contrast, the other two patients experienced no decline in renal function and showed no change in MAG3 renograms. There were no consistent changes in urinary RBP in any of these patients (Table 3).

### Hyperglycaemia

Seliciclib-induced hyperglycaemia was observed in five patients at 800 mg b.i.d., but reached grade 3 in only one patient with associated glycosuria. This patient was not receiving concurrent corticosteroids. The hyperglycaemia was transitory, being noted by the end of the treatment period on day 7 and fully normalised on cessation of drug therapy.

### Other non-haematological toxicity

One patient at the 800 mg dose level experienced a grade 3 rash occurring on day 7 of the first treatment cycle. This was characterised by an intensely pruritic and erythematous, urticarial rash of diffuse distribution affecting the trunk, lower limbs and arms associated with mild systemic upset and pyrexia. The patient was treated with topical steroid creams, emollients and antihistamines, and the rash resolved. The patient was subsequently rechallenged with a second cycle of treatment and within 4 h of the first seliciclib dose, the rash reappeared. The patient was commenced on oral steroids and the rash again resolved within 2 weeks. Subsequent expert dermatological review confirmed that these episodes were likely to be drug related, and were of a vasculitic appearance. Associated biochemical abnormalities in the same patient were also noted on day 7 of the treatment period during both cycles 1 and 2, namely hypokalaemia, raised urea and creatinine and hyperglycaemia. These values were within normal limits before treatment and resolved within days of finishing seliciclib.

Derangements in liver biochemistry, principally, elevations of *γ*GT and ALP were also reported during the 7-day treatment period at both 200 mg b.i.d. (4 out of 6 patients) and 800 mg b.i.d. (10 out of 12) dose levels. Most of the derangement was mild to moderate. Raised liver biochemistry tests were seen by day 7 and were reversible at the end of the course with the exception of the DLT at the 200 mg dose level, which was probably tumour related.

### Pharmacokinetics

**Figure 2:**
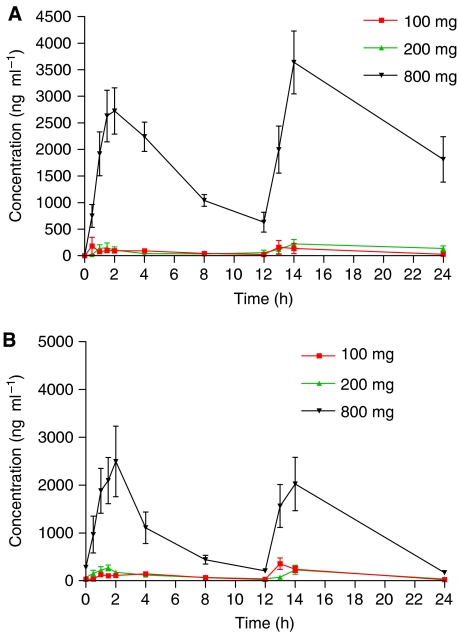
Concentration–time pharmacokinetic profiles (mean±standard deviation) for all the evaluated dose levels of seliciclib (100, 200, 800 mg) on both days 1 (**A**) and 7 (**B**).

**Figure 3:**
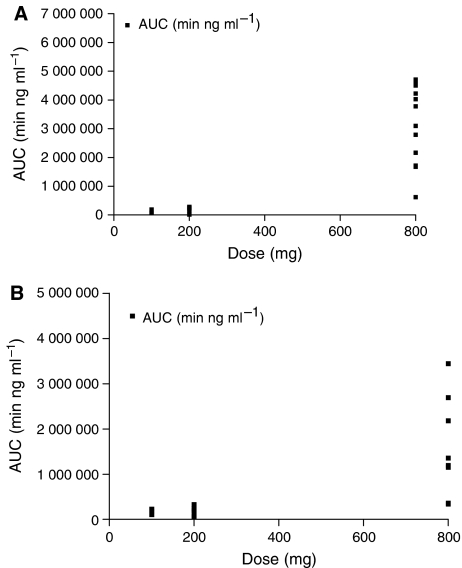
Seliciclib AUC_(last)_ values for all tested dose levels, on both days 1 (**A**) and 7 (**B**), as a function of daily administered dose.

**Figure 4:**
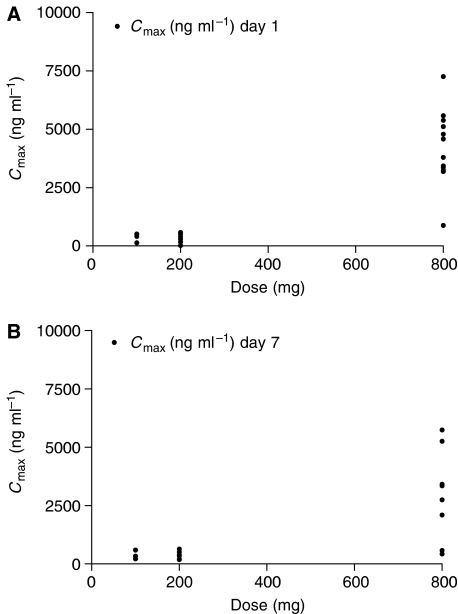
Seliciclib *C*_max_ values for all tested dose levels, on both days 1 (**A**) and 7 (**B**), as a function of daily administered dose.

The mean concentration vs time profile for each dose level is shown in Figure 2. Seliciclib was absorbed relatively slowly with a mean *T*_max_ of 2.55 h (range 1–4 h). The plasma clearance was greater than liver blood flow and the terminal half-life was similar in all dose groups (2–5 h). Table 4 summarises the pharmacokinetic parameters for patients treated with seliciclib at each dose level. Area-under-the-curve (AUC) and *C*_max_ of seliciclib increased with dose (Figures 3 and 4). There is a suggestion of nonlinearity, but this is unclear from the limited data generated by this study. There were no significant differences in the PK parameters calculated on days 1 and 7. The ratio of the carboxylic acid derivative which is the major metabolite (De la Motte and Gianella-Borradori, 2004; Nutley *et al*, 2005) to the parent compound, varied from 0.8 to 43 (data not shown). However, there was no correlation between the ratio of metabolite to parent and the plasma clearance of the parent drug. The mean *C*_max_ at the 800 mg dose level (3 mg ml^−1^, 10 *μ*M) was in the range of IC_50_ values reported for seliciclib *in vitro* activity (McClue *et al*, 2002; Nutley *et al*, 2005; Raynaud *et al*, 2005), but this concentration was not sustained for the period required to exert an antitumour effect *in vitro*.

### Pharmacodynamics

Retinoblastoma phosphorylation and cyclin D1 levels were studied in peripheral blood mononuclear cells, compared with appropriate controls. No reliably detectable alterations were observed. Tumour biopsies were not obtained in this study.

### Antitumour activity

No objective tumour responses were observed. One patient with metastatic ovarian cancer, whose disease was progressing before treatment with seliciclib, had disease stabilisation for a total of six courses, with no change in her CA-125 tumour marker. Two of three patients at the 100 mg b.i.d. dose level, two of six at the 200 mg b.i.d. dose level and three of 12 patients at the 800 mg b.i.d. dose level had stable disease after course 2, but progressed by course 4.

## DISCUSSION

Cell-cycle dysregulation is a hallmark of malignancy. The Cdks play a crucial role in controlling progression through the cell cycle, and genetic and epigenetic mechanisms frequently result in their deranged expression and/or activity in oncogenesis. The development of Cdk inhibition has therefore been pursued as a potential therapeutic strategy, although the development of selective inhibitors of this serine–threonine kinase family has been particularly challenging in view of the high degrees of sequence homology between Cdks and other kinases. It has been reported that Cdk inhibitors could inhibit tumour cell growth in preclinical models, but many questions remain about which Cdks should be targeted for anticancer therapy. These concerns have been fuelled by RNA interference and knockout mice studies, indicating that functional redundancy may exist between different Cdks, with the loss of Cdk2 alone failing to inhibit tumour cell proliferation (Ortega *et al*, 2003; Tetsu and McCormick, 2003), and the loss of both Cdk4 and Cdk6 not preventing cell cycling (Malumbres *et al*, 2004). These data suggest that the optimal pharmacologic Cdk inhibitor should selectively inhibit a broad spectrum of Cdks, including Cdk1, 2, 4, 6, yet spare other kinases whose inhibition results in nonspecific toxicity (Sausville, 2002).

Seliciclib is an orally bioavailable selective inhibitor of Cdk 1, 2, 7 and 9 (McClue *et al*, 2002; Whittaker *et al*, 2004). Cassette dosing PK studies indicated that seliciclib had the most favourable PK profile of a library of 107 different 2,6,9-tri-substituted purine Cdk2 inhibitors, prepared by parallel synthesis (Raynaud *et al*, 2004). Seliciclib showed promising activity in preclinical models, and decreased both RB and RNA Pol II phosphorylation and total RNA Pol II protein, suggesting that in addition to Cdk2 inhibition, it also inhibits transcription, possibly via inhibition of Cdk7 and Cdk9 (McClue *et al*, 2002; Whittaker *et al*, 2004; MacCallum *et al*, 2005; Raynaud *et al*, 2005). Similarly, seliciclib also decreases cyclin D1 protein levels independently of the p38SAPK and phosphatidylinositol 3-kinase pathways and causes a reduction in the expression of cyclins D1, A and B1, presumably by the same mechanism. Its cell-cycle effects include a reduction of cells in G_1_, inhibition of bromodeoxyuridine incorporation during S phase and a moderate increase in G_2_/M phase.

We now report the clinical evaluation of the orally bioavailable selective Cdk inhibitor seleciclib in cancer patients, using oral dosing b.i.d. for 7 days every 3 weeks. Preclinical toxicology studies with 28 days treatment in rodents reported anaemia, leucocytosis, bone marrow hyperplasia, polydipsia, polyuria and gastric ulceration. Preclinical data indicated that exposure to seliciclib in the concentration range 7.9–30.2 *μ*M over a 24-h period could achieve clinically relevant biological effects (De la Motte and Gianella-Borradori, 2004; Whittaker *et al*, 2004; Tang *et al*, 2005). *In vivo* studies also indicated that a single dose of 500 mg kg^−1^ (2750 mg m^−2^ of mouse body surface area) achieved levels of ≥10 *μ*M for 24 h (Raynaud *et al*, 2005). In men, a single dose of 250 mg m^−2^ (400 mg dose for a patient with a body surface area of 1.8 m^2^) was predicted to achieve the same level for 4 h. However, in a single dose bioavailability study in healthy volunteers, considerable interindividual variability in drug exposure was observed, with most of the drug being cleared by 12 h (De la Motte and Gianella-Borradori, 2004).

In this phase I trial, no significant drug-related toxicity was observed at the 100 or 200 mg b.i.d. dose levels, and PK data suggested that these doses resulted in low drug exposure (see Figure 3), hence the dose was increased to 800 mg b.i.d. for 7 days. At this level, dose-limiting toxicities were reported comprising reversible hypokalaemia, hyponatraemia, elevated *γ*GT, hyperglycaemia and a generalised vasculitic skin rash. In addition, a rapid rise in creatinine during the administration period was also frequently observed. Although this did not reach grade 3 and appeared to be reversible on stopping the drug, it was unexpected and of concern in the absence of a clear explanation. Similar toxicities have been reported in a separate phase I study utilising a 5-day, twice-daily administration, oral schedule (Pierga *et al*, 2003). In that study, vomiting, skin rash, hypokalaemia and raised creatinine were also seen. The recommended phase II dose in that study was 2500 mg per day for 5 days, although this was reported to be associated with manageable grade 3 hypokalaemia and grade 3 skin rash. Preclinical toxicology had reported seliciclib-related polyuria and polydypsia, but not renal dysfunction or hypokalaemia.

The pathogenesis of the creatinine rise is not fully understood, but may be associated with a reversible reduction in renal blood flow. Some evidence for this was obtained from the serial evaluation of MAG3 isotope renograms that detected a significant, reversible, decrease in renal blood flow in one patient, following seliciclib therapy. The pathogenesis of this possible alteration in renal blood flow remains unexplained. It has been proposed that binding of seliciclib to unrelated targets such as adenosine receptors, which regulate renal blood flow, could explain these findings, but this has not been confirmed to date (Benson *et al*, 2005). The reversibility of the renal dysfunction and absence of changes in urinary retinol-binding protein do, however, suggest that this was not related to clinically significant tubular damage.

Although clearly distinct from the renal dysfunction, in that the two events did not always occur simultaneously, reversible dose-limiting hypokalemia was also observed during this study. Whereas this was noted at the 200 mg dose level, potassium levels below 3.0 mmol l^−1^ were only observed at 800 mg, with potassium levels as low as 2.2 mmol l^−1^ in one patient. The hypokalaemia was easily and rapidly reversible with potassium supplementation and on discontinuation of seliciclib dosing. It was, however, thought to be potentially dangerous and would warrant very close monitoring. The pathogenesis of the hypokalaemia has not been elucidated. As renal collecting duct and tubular reabsorption of potassium (involving carbonic anhydrase) is critical to potassium homeostasis, these processes may be implicated. Further studies on the effects of seliciclib on adenosine receptors, carbonic anhydrase and renal collecting duct and tubular function may be useful.

The pharmacokinetic data indicate that seliciclib levels were not maintained at the level associated with antitumour activity in some xenograft models. The mean maximum plasma concentration achieved at the highest dose level was approximately 3 *μ*g ml^−1^ (10 *μ*M), whereas in the mouse, a peak seliciclib concentration of approximately 100 *μ*M and nearly 24 h of exposure to >10 *μ*M was achieved with a single dose of seliciclib at 500 mg kg^−1^ p.o. When administered b.i.d., this resulted in significant growth delay of a human tumour xenograft (Raynaud *et al*, 2005). However, the lowest effective dose in this xenograft model was not established. More sensitive tumours may not require such high drug levels and it is unclear whether this discrepancy in drug exposure was responsible for the failure to demonstrate an effect on RB phosphorylation and cyclin D1 in peripheral blood mononuclear cells. A number of other factors may have contributed; for example, peripheral blood mononuclear cells may be inappropriate as a surrogate tissue for these agents in man, as they are not actively dividing. Buccal mucosa scrapes have been used to demonstrate changes in p53 and phospho-RB in response to flavopiridol, although this did not translate into detectable changes in tumour biopsies (Tan *et al*, 2004). In a separate study with flavopiridol, flow cytometric studies to examine cell cycle changes and apoptosis in peripheral blood mononuclear cells were uninformative (Thomas *et al*, 2002).

Side effects such as neutropenia and gastrointestinal toxicity, such as have been reported with other Cdk inhibitors, were not observed during this study. It is not entirely clear what side effects are to be expected from a broad-spectrum Cdk inhibitor, such as seliciclib. If the biologically important effects of seliciclib are on transcriptional regulation, it is hard to predict exactly what pattern of side effects is likely to be observed with this agent. The observation of seliciclib-related hyperglycaemia in five of 12 patients at the 800 mg dose level is interesting. This has been reported with other Cdk inhibitors, possibly through inhibition of Cdk5, which has a role in regulating insulin secretion (Sausville, 2002).

In this study DLT was observed at 800 mg, given b.i.d. for 7 days every 21 days. Although the toxicities were reversible, they were nevertheless of concern and it proved difficult to continue dosing for 7 days in some patients in the presence of a consistently rising creatinine. Plasma concentrations were not maintained above the average IC_50_ concentration at this dose and schedule. However, shorter duration regimens, that is, b.i.d. administration for 5 days, have proved to be feasible and permit administration of higher daily doses (Pierga *et al*, 2003). It was the decision of the sponsor to pursue clinical development of the shorter duration regimen and not to continue further investigation of the 7-day continuous dosing regimen. Further investigations are required to explain fully the renal side effects observed with seliciclib. An orally bioavailable Cdk inhibitor such as seliciclib has significant attractions. The development of alternative PD biomarkers will assist the identification of the optimum dose and schedule.

## Figures and Tables

**Table 1:** Patient characteristics

*Gender*	
Male	15
Female	6
Median Age (range) years	62 (39–73)
	
*Performance status*	
0	2
1	17
2	2
	
*Site of primary disease*	
Colorectal	9
Ovarian	2
Unknown primary	2
Lung	1
Cervical	1
Prostate	1
Other	5
	
*Prior treatment regimens*	
Chemotherapy	
1	6
2	9
3	7
3+	1
Prior hormone therapy	1
Prior radiation	8
	
No. of cycles of seliciclib administered	42
Median per patient	2
Range	(1–6)

**Table 2:** Reported toxicity profile during the first course of seliciclib treatment

	**Seliciclib 100 mg b.i.d**	**Seliciclib 200 mg b.i.d**	**Seliciclib 800 mg b.i.d**
No. of subjects	3	6	12
No. with grade 3 & 4 adverse events	0	2	7
*γ* GT increase	0	1	3
Hypokalemia	0	0	3
Hyperglycaemia	0	0	1
Hyponatraemia	0	0	1
Rash	0	0	1
Hypotension	0	0	1
Lymphopenia	0	0	1
Anorexia	0	0	1
Fatigue	0	0	1
ALT increased	0	1	0
AST increased	0	1	0
Alk Phos increase	0	1	0
Bilirubin increase	0	1	0
CKMB increase	0	1	0

**Table 3:** Urinary retinol binding protein levels following treatment with seliciclib

	**Urinary retinol binding protein (0.10–100.0 mg/mol creatinine)**		
	**Patient 20**	**Patient 21**	**Patient 22**
Pretreatment	8.7	136.4	6.8
Day 3	59.2	44.7	7.9
Day 14	20.8	25.7	40.1

**Table 4:** Summary of Seliciclib pharmacokinetics during course 1

	**Dose**		***C*_max_ (ng ml^−1^)**	***T*_max_ (min)**	***t*_1/2_ (min)**	**AUC_last_ (min ng ml^−1^)**	**AUC_inf_ (min ng ml^−1^)**
Day 1	100 mg (*n*=3)	Mean	248	112	267	112236	115384
		%CV	91	101	55	58	56
		Median	134	61	278	82403	90289
	200 mg (*n*=6)	Mean	179	279	176	165949	181877
		%CV	123	95	42	62	59
		Median	80	167	149	185025	230597
	800 mg (*n*=12)	Mean	3197	177	219	3163604	3177921
		%CV	42	68	21	43	43
		Median	3161	120	232	3441798	3452569
							
Day 7	100 mg (*n*=3)	Mean	162	190	268	171555	173422
		%CV	15	46	24	36	35
		Median	152	240	234	181815	182620
	200 mg (*n*=6)	Mean	310	62	781	175903	255297
		%CV	43	71	173	59	89
		Median	303	60	228	184620	208748
	800 mg (*n*=8)	Mean	2774	99	230	1590950	1598426
		%CV	71	26	43	69	69
		Median	2636	107	209	1275901	1278974
